# Geospatial and Temporal Inventory for Industrial DDT Waste Disposal to a Deep Coastal Ocean Environment

**DOI:** 10.1021/acs.est.5c03851

**Published:** 2025-09-18

**Authors:** Mong Sin Christine Wu, Jacob T. Schmidt, Hailie E. Kittner, Earth 182B Group, David L. Valentine

**Affiliations:** † Department of Earth Science, University of California, Santa Barbara, California 93106, United States; ‡ Interdepartmental Graduate Program in Marine Science, University of California, Santa Barbara, California 93106, United States; § Marine Science Institute, University of California, Santa Barbara, California 93106, United States

**Keywords:** Chlorinated petrochemicals, deep ocean dumping, legacy pollution, pesticide use, San Pedro Basin, contaminant fate and transport, temporal deposition

## Abstract

Historical ocean disposal of industrial DDT (dichloro-diphenyl-trichloroethane) waste off Southern California is implicated in extensive ecological impacts extending well beyond this region, but details around disposal, transport, and fate are poorly characterized for deep ocean settings such as this. Here, we present results from an 814 km^2^ survey of a primary disposal area, San Pedro Basin, by mapping the spatial distribution and depositional history of DDT and its immediate daughter products DDD (dichloro-diphenyl-dichloroethane) and DDE (dichloro-diphenyl-dichloroethylene). We find highly elevated concentrations for all three compounds throughout the study area, with “hotspots” from primary deposition and secondary migration processes. The majority of DDT and DDD is buried within a thin sediment layer consistent with peak deposition in the 1950s, whereas substantial amounts of DDE still linger in overlying sediments with an apparent contribution from the Palos Verdes Shelf. We characterize intense spatial variability across distance scales and develop an approach to address the resulting uncertainty toward estimating a total modern burden of ∼30–36 tonnes (DDT, DDD and DDE) in this area. These results are foundational for informing DDT’s deep-sea transport and transformation processes and for defining the linkage of offshore disposal to current ecological problems in this region and beyond.

## Graphical abstract

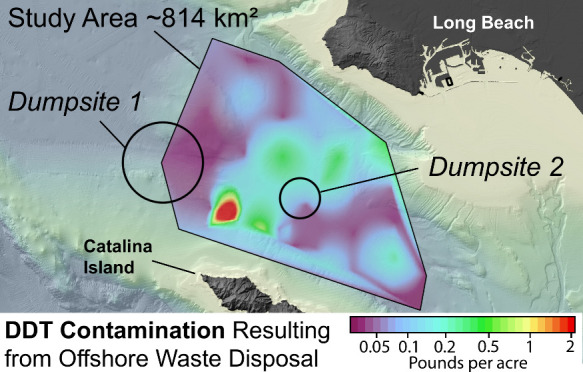

## 1 Introduction

Considered a wonder pesticide in the 1940s and 1950s, technical DDT (comprised mainly of 4,4′-dichlorodiphenyltrichloroethane) fell out of favor following the publication of Rachel Carson’s Silent Spring,^1^ and was banned for agricultural use in the United States (US) in 1972. Research has since shown that DDT exposure contributes to environmental and human health problems that include egg-shell thinning in birds,^2,3^ cancers in humans^4^ and sea lions,^5^ endocrine disruption in dolphins,^6^ and multigenerational health effects in humans.^7,8^ Bans on technical DDT use have expanded since the initial US ban, culminating in a worldwide ban except for disease vector control by the Stockholm Convention on Persistent Organic Pollutants in 2004. Yet, DDT and its breakdown products (including DDD and DDE; see full names in Table S1) persist in the environment today.^9–11^

During the period of active production in the US, the nation’s largest DDT manufacturing facility – operated by Montrose Chemical Corporation of California (Montrose) – was located in Torrance, California. Montrose synthesized technical DDT at their facility in Torrance beginning in 1947, producing an estimated 1–8 million pounds per month prior to the shuttering of the facility in 1982.^12^ Montrose’s synthesis involved the reaction of chloral with chlorobenzene in strong sulfuric acid and generated two substantial streams of acid waste that were disposed directly or indirectly to the Pacific Ocean.^12^

Starting in 1947 Montrose disposed of their reactor waste – including residual reactants, unrecovered products and byproducts in strong sulfuric acid – through bulk offshore disposal.^12^ By this approach, waste was picked up by a disposal service known as California Salvage Company (Cal Salvage), trucked to Berth 115 in the Port of Los Angeles, transferred to a tank barge, and later towed offshore where it was pumped directly from the tank barge into the surface water of the San Pedro Channel.^9,13,14^ Though records are limited and permits were issued retroactively, disposal of Montrose’s acid waste is thought to have been centered at either or both of two locations known as dumpsites 1 and 2 (Figure 1). This practice allegedly ceased after 1960, when an acid recycling unit was constructed at the Montrose facility.^12^

**Figure 1:**
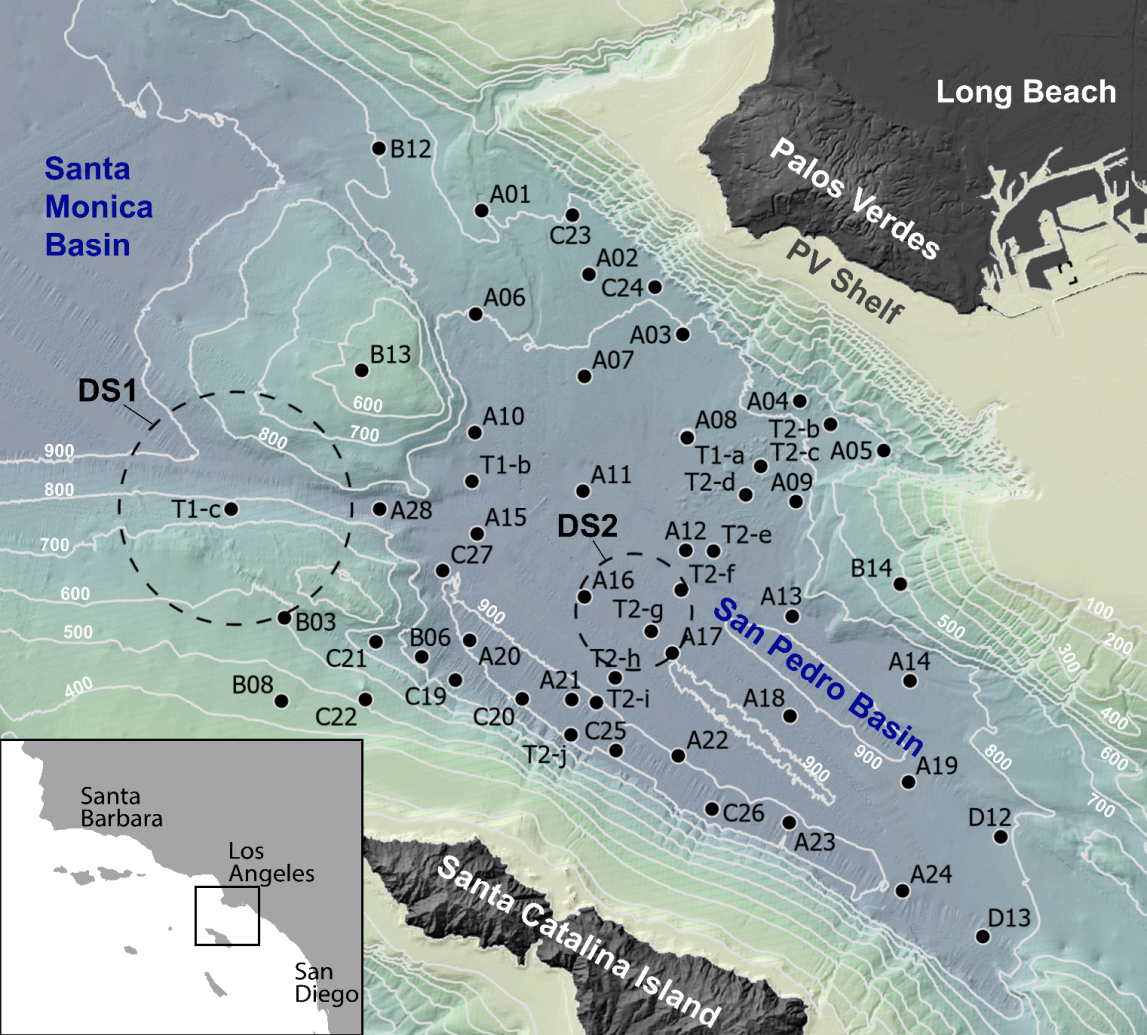
Bathymetry map showing the 54 sediment core sampling locations across San Pedro Basin. Color and contours indicate water depth. Inset shows the location of the study area (black box) along the Southern California coastline. Dashed circles indicate the locations of ocean disposal sites referred to as “dumpsite 1” (DS1)^27^ and “dumpsite 2” (DS2).^28^

Montrose also disposed of neutralized acidic and caustic rinsate into the sanitary sewer that was ultimately discharged to the shallow coastal waters of Palos Verdes Shelf (PVS). Prior to 1953, Montrose washed technical-grade DDT product twice to remove residual sulfuric acid and chlorobenzene; after both washes, dilute sulfuric acid wastes were neutralized with sugar lime and drained to an on-site wastewater pond and then to the sewer.^12^ After 1953, technical-grade DDT product was statically separated from residual sulfuric acid then washed with 15% NaOH. Both acid and caustic wastes were drained to below-ground tanks then neutralized before discharge into the sewer.^12^ Discharge to PVS through the sewer effectively ceased in 1970 following detection of chlorobenzene and technical DDT in Montrose sewage discharge.^12,15^ Litigation over the discharge ultimately led to a settlement that included Superfund status for PVS and broad language encompassing ‘offshore areas,’ which were defined to include “any ocean dumpsites used for disposing wastes from the Montrose Property Plant and any offshore areas to which hazardous substances, including without limitation DDT, aerially or otherwise originating from the Montrose Plant Property or the Stauffer Dominguez Plant Property have or may come to be located.”^16^

Substantial efforts associated with litigation have characterized the discharge of DDT waste to the shallow waters of PVS,^17–19^ with discharge estimates of 870–1450 tonnes^20^ and sediment burden estimates of 110–132 tonnes for PVS sediments.^18,21^ Much less is known about the wastes disposed offshore. Select offshore sediment samples have been observed to host high concentrations of DDT and its metabolites (DDT, DDD and DDE are collectively referred to here as DDX).^22–24^ Recent research^9^ has shown that 1950s-era offshore disposal distributed DDT and its breakdown products along a transect spanning the width of San Pedro Basin (SPB) – from PVS to the Catalina Island Rise. Deep dwelling fish in the region have also been found to contain DDT-related compounds in their tissues, potentially from offshore disposal.^25,26^ Still, major uncertainties remain – including the quantity, location, disposition and impacts of this offshore disposal activity as well as broader questions about transport and fate of DDT waste in deep ocean environments.

In this work we describe the results of a sampling campaign focused on quantification of DDX in sediment from an 814 km^2^ area encompassing SPB. From these results we are able to estimate the modern DDT and DDX burden for sediments of SPB, determine the spatial and temporal patterns of deposition and degradation, and identify scales of spatial variability – all of which are foundational for assessing fate, transport and ecological impacts in this region and elsewhere.

## 2 Materials and Methods

### 2.1 Sediment Sampling, Treatment, and Instrumental Analysis

Sediment from SPB, California, was collected through sediment coring during multiple expeditions as follows: R/V *Yellowfin* (A-series, March 6–8, 2023; C-series, July 24–28, 2023; and D-series, August 5–9, 2024) and R/V *Atlantis* (B-series, June 24–July 11, 2023). Note that we further incorporate published results from Schmidt et al., 2024,^9^ collected November 07 and December 15, 2022 from the R/V *Yellowfin* (T-series). Sediments were collected in a grid pattern by sediment multicorer (MiniMuc, K.U.M., Kiel, Germany) in March 2023, with additional cores taken during the other reported sampling events (B-series, MC-400, Oregon State University; all others, MiniMuc, K.U.M., Kiel, Germany). In total, we report results from cores collected at 44 stations with 568 unique samples (Figure 1) in addition to the 10 stations (and 146 unique samples) reported previously by Schmidt et al. (2024).^9^

Sediment core samples were processed using two distinct methods: as 1 or 2 cm horizontal slices, and as 12 cm vertically integrated subcores. Horizontally sliced cores were collected at every station and processed shipboard as described previously, with processing equipment rinsed between samples with isopropyl alcohol (70% v/v).^9^ Vertically integrated subcore samples, also referred to here as mini-piston cores, were collected in triplicate only in March 2023 for the A-series stations using 50 cc. borosilicate glass syringes cut to 12 cm length. The three syringe barrels were inserted sequentially into the surface of the sediment core to 12 cm depth while the plunger was held constant relative to the sediment surface, arranged adjacent to one another in a triangular formation. Syringes were then withdrawn and the contents extruded into individual 125 mL glass jars with PTFE-lined closures and stored at −20 °C until used for chemical analysis. For sampling stations located close to PVS, a second set of vertically integrated subcores was collected as well, between 12–24 cm sediment depth, to capture DDX that may be buried deeper than 12 cm as previously observed at T2-b, a station immediately adjacent to PVS.^9^ Sampling was performed in triplicate by repeating the procedure above after removing residual sediment material in the 0–12 cm depth interval. Between depth intervals and cores, subcoring equipment was cleaned of sediment particles, rinsed with surface seawater then isopropyl alcohol (70% v/v), and dried. For station A09, the 12–24 cm subcore was not collected at the time of sampling, hence this sample was later reconstituted in the laboratory by combining equal wet weight mass of horizontal slice samples of that depth interval and homogenizing thoroughly.

For each relevant station, both horizontally sliced and vertically integrated subcore samples were extracted and cleaned following EPA methods 3570, 3630, and 3660 then analyzed for pesticides content by GC-ECD following EPA method 8081B at Pace Analytical (previously Alpha Analytical), Mansfield, Massachusetts as described previously.^9^ Prior to analysis, all samples were homogenized twice. Depth distributions of DDT, DDD and DDE for each core are provided in Figure S1 with the corresponding data provided in the data supplement.

### 2.2 Data Analysis

#### 2.2.1 Sediment Age Model

In order to explore how the spatial patterns of sediment DDX deposition have varied in time, we synchronized the chronology for all SPB sediment cores based on the depth of the peak DDT layer. This approach is based on the assumption that the peak deposition of industrial DDT into SPB was contemporaneously preserved in sediments throughout the deep basin floor. This assumption is strongly supported by our prior findings^9^ based on radiocarbon, ^210^Pb and ^137^Cs chronologies for several depth-resolved cores also included in this work. Those results determined peak DDT burial around 1955, which is corroborated by historical records.^9^ Based on this approach and our prior findings we then developed simple age models for each depth-resolved core by linearly interpolating sediment age between year 1955 at the depth of peak DDT concentration and year of sampling at sediment surface. We also extrapolated to older intervals based on the same relationship, with the caveat that DDT found (at relatively low concentration) in older sediments was presumably mixed there through physical or biological processes and is not autochthonous to that strata. Basin scale maps of sediment accumulation relative to select chemical strata are provided in Figure S2.

#### 2.2.2 Spatial Interpolation

To visualize the spatial patterns of DDX across SPB, we created maps using a natural neighbor spatial interpolation method to estimate the values in between measured points. The natural neighbor interpolation method interpolates values at query points based on measured values at the closest subset of input sample points that are weighted on proportionate areas of Voronoi cells.^29^ This method is ideal for our study given the unevenly distributed sampling locations, as this method has the advantage of being an exact interpolator that creates estimated surfaces that are smooth and highly adaptable to local variations.^29^ Unlike some other commonly used methods such as inverse distance weighting and Kriging, natural neighbor interpolation is also free of the choice of parameters and geostatistical model assumptions, leading to more unbiased results.

#### 2.2.3 Estimation of Total DDX Burden

The total burden of DDX was estimated for the 814 km^2^ study area using the abundance measured for each core. Based on the DDX concentrations measured in each depth-resolved slice, we first calculated a vertically integrated average DDX abundance for the top 30 cm of each sediment core by estimating values at depths where measurements were not made using linear interpolation/extrapolation from closest depths. Since most of the unmeasured depths are downcore from the peak DDX horizons and bracketed by low DDX concentrations, uncertainties from such samples have very little impact on the overall burden estimated for the entire core. We then calculated the top 30 cm DDX inventory of each core on a per-unit-area basis using site-specific % total solids measurements and basin-wide dry sediment density of 2.5832 ± 0.0049 g/cm^3^ of a composite sediment sample taken from SPB (See Supporting Information S1). We then spatially interpolated the inventory values calculated at each station over our entire study area using a natural neighbor interpolation method to estimate an overall DDX burden of the top 30 cm sediment.

#### 2.2.4 Monte Carlo Iterations of DDX Burden Estimation

To evaluate our confidence in the estimation of the overall DDX burden, we employed two distinct Monte Carlo-based approaches. Our first approach aims at assessing the uncertainty of the overall DDX burden estimate based on the depth-resolved sediment slice samples as described in the previous section, whereas our second approach evaluates how small-scale variability in DDX concentrations may influence the overall burden estimation.

In the first approach, we employed a combination of jackknife and bootstrap methods to our spatial data. First, we applied a jackknife method to randomly assign our sample stations into N distinct, nonoverlapping subsets. Each subset yields a DDX burden estimation (using natural neighbor interpolation), from which we calculated the average and standard deviation among these N burden estimates. Second, we applied bootstrap to the previous step by repeating the step 1000 times. This results in 1000 iterations of average burden and uncertainty estimates for that particular value of N. Third, we systematically performed the previous steps for N equals 2 to 9, that is, we jackknifed our sample stations into 2, 3, 4, ...., and 9 subgroups. Last, since estimates from each N represent the statistical power of 1/N of the sample stations, we could evaluate the trend of how uncertainty changes with respect to N, and extrapolate this trend to estimate the uncertainty for N = 1, which represents the uncertainty in burden estimate from all of the sample stations.

In the second approach, we incorporated data from the mini-piston core triplicates to compute an uncertainty distribution of the top 30 cm DDX inventory estimated at each station. For the A-series stations where mini-piston core samples were collected, we treated the vertically integrated depth-resolved core, and the mini-piston core triplicates, as four separate measurements at each site. A weighted mean and weighted standard deviation of inventory estimation was then calculated using their cross-section areas as weights. Since mini-piston core samples were collected only for 0–12 cm at most sites and also 12–24 cm at a few sites, we assumed a consistent proportion of DDX among 0–12 cm, 12–24 cm, and 24–30 cm depth intervals, using the depth-resolved concentration profile as a reference. Using this proportionality we scaled the DDX for each mini-piston core to account for DDX residing beneath the reach of the sampling approach when we computed the mini-piston-core-based DDX inventory estimations. For the other stations where mini-piston cores were not collected, we assigned an uncertainty standard deviation to each station based on the average 56.5% standard deviation calculated from all A-series stations. We then performed 1000 Monte Carlo iterations, with each iteration randomly sampling a site-specific inventory estimation from the normal distribution (mean and standard deviation) at each of the 54 sites. 1000 natural neighbor spatial interpolations of DDX inventory estimations were then generated, which allowed us to evaluate the mean and uncertainty of our basin-scale DDX burden estimation.

## 3 Results and Discussion

### 3.1 Spatial and Temporal Patterns of DDX Deposition

The patterns of preservation for DDT, DDD and DDE in the deep SPB each exhibit remarkable coherence with respect to location within the basin (Figure 2) and stratigraphic position within the seabed (Figure 3). Observed patterns are useful indicators for the deposition, transport and preservation controls at the basin-scale, with key caveats. Notably, DDT may undergo a dechlorination cascade through biotic and abiotic routes, leading to daughter products with 4, 3, or 2 chlorine atoms.^20,30,31^ In this work we quantified the two common structural isomers of DDT (4,4′ and 2,4′) along with the four daughter products commonly produced as a result of a single chlorine elimination (2,4′ and 4,4′ DDE and DDD). Further dechlorination products were not assessed by our method but are likely important toward greater understanding of mass balance and transport.

**Figure 2:**
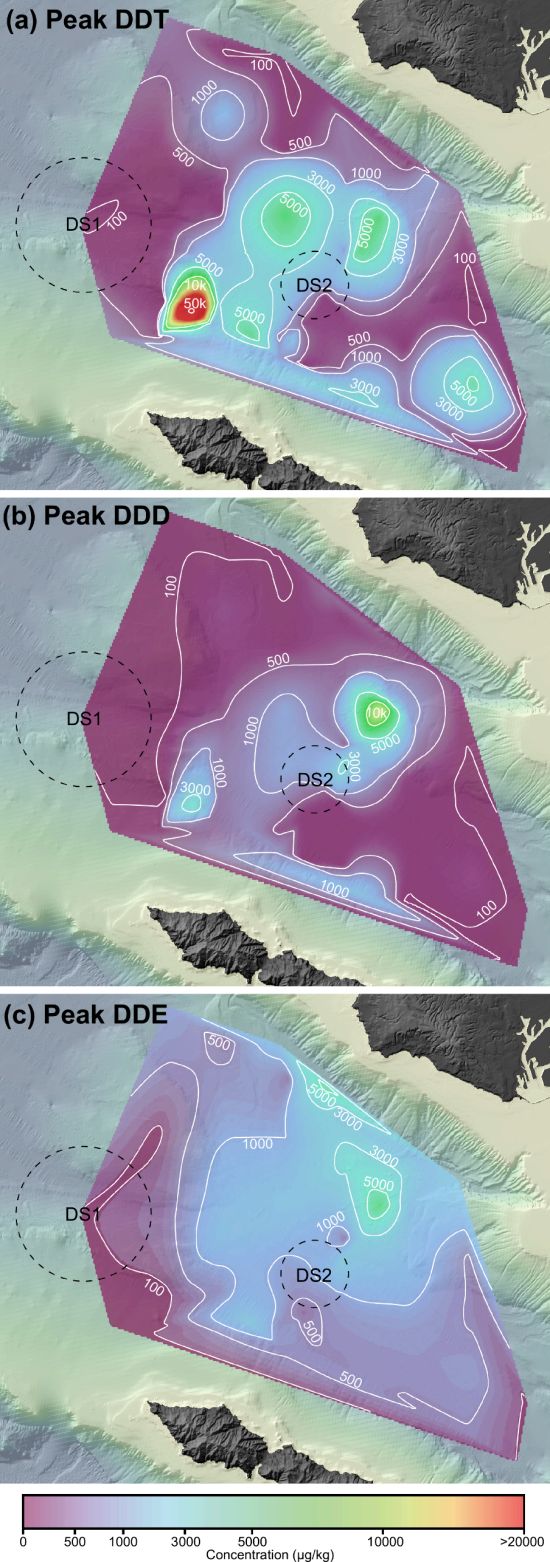
Spatial distribution of (a) peak DDT, (b) peak DDD, and (c) peak DDE concentrations buried in sediments across San Pedro Basin. Contours show concentration in μg/kg dry weight of sediment. Note that the color scale is nonlinear and skewed toward the lower end. Dashed circles indicate the locations of dumpsite 1 (DS1) and dumpsite 2 (DS2).

**Figure 3:**
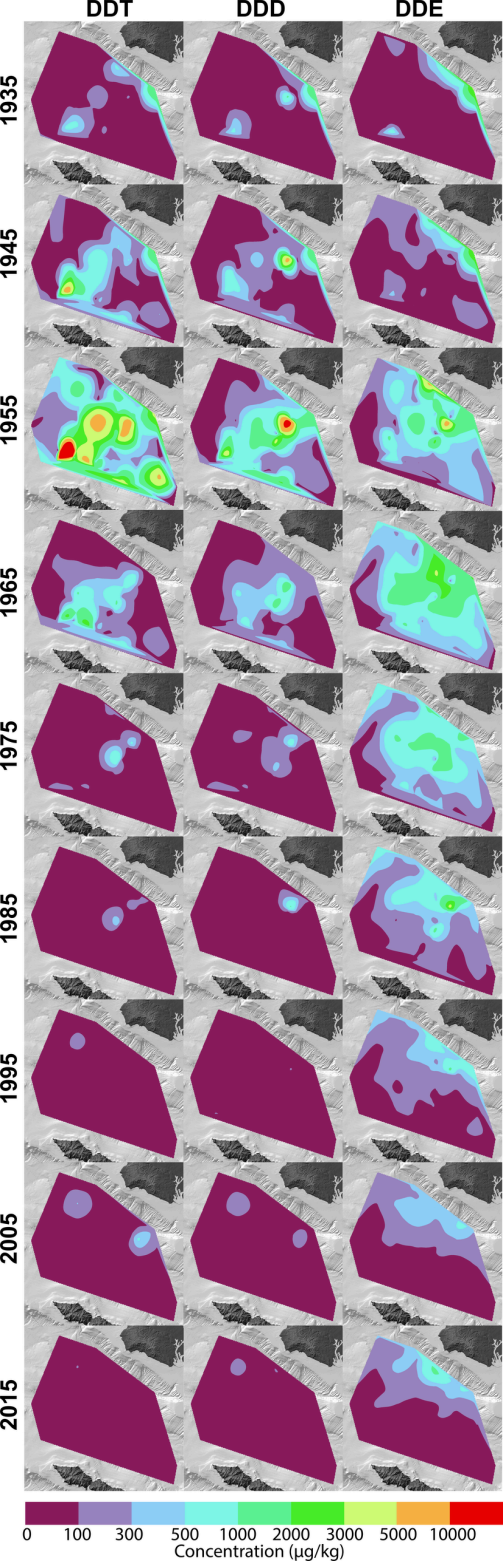
Decadal time-series of spatial distributions of DDT (left), DDD (middle), and DDE (right) concentrations.

Peak DDT concentrations within all vertically resolved sediment core profiles range from 4–53,600 μg/kg at our sites with a maximum at 6–8 cm for most cores located distal to the mainland (Figure 2a; Figure S2). A NE-SW band of high DDT concentrations (>1000 μg/kg) spans SPB, with a distinctive hotspot of DDT reaching >50,000 μg/kg located at Station B06 in the westernmost periphery of SPB off the northernmost coast of Catalina Island. Peak DDD concentrations vary between 11–17,650 μg/kg and show a NE-SW band of high concentrations across SPB similar to that of DDT (Figure 2b). Peak DDE concentrations, however, show a distinct spatial pattern compared to DDT and DDD. While DDE also shows an elevated concentration band extending from NE to SW across the basin, the highest concentrations are found along the basin’s northern edge, adjacent to PVS (Figure 2c). These patterns are unexpected because the concentrations for DDT and DDE show no clear association with dumpsite 2, instead exhibiting elevated concentrations throughout SPB.

Synchronized age models of all sediment cores using the peak DDT horizon as an age marker (see Section 2.2) allow us to further reconstruct the history of DDT, DDD and DDE deposition and transformation across SPB. Heatmaps of decadal time steps (Figure 3, Supplemental Video) using vertically integrated concentration data pin peak DDT deposition to the era of offshore disposal, which is supported by the emergence of continuity with elevated concentrations spanning the central SPB, as well as by published chronology.^9^ Lower concentrations of DDT in overlying sediments suggest a sharp decrease in offshore waste deposition following the era of peak disposal. The spatiotemporal pattern for DDD mimics that of DDT, consistent with *in situ* reductive dechlorination of DDT to DDD as the primary source^31,32^ rather than direct deposition of DDD or transport from PVS. In contrast, DDE exhibits a distinct spatiotemporal pattern. While DDE concentrations increase abruptly in the 1950s similar to DDT, peak DDE concentrations sustain into the 1960s, and only gradually decrease in later decades. Substantial DDE is present in the recent surficial strata dating to the 2010s, particularly in the deep SPB adjacent to PVS, which still hosts substantial DDE contamination.^33^

Several key issues emerge from the observed spatiotemporal patterns of DDT, DDD and DDE that warrant mention. First, DDT, DDD and DDE are observed in strata assigned to the 1940′s, prior to industrial DDT manufacturing. We attribute this to minor amounts of bioturbation in the sediments, minor relief in the surface texture of sediment that can blend adjacent strata at subcentimeter scale, and/or diffusive flux. Second, it remains unclear why DDT was so effectively preserved from offshore disposal, whereas subsequent deposition was primarily in the form of daughter product DDE. We suspect this relates to the low oxygen concentration in and settling process to the deep SPB, contrasting with biogeochemical activity associated with resuspension and or protracted transport from the more oxygenated PVS. Third, observed distributions for DDT, DDD and DDE inform physical transport mechanisms within the water column. Specifically, the basin-wide distribution of DDT and DDD suggests dispersion in the water column occurred coincident with settling for bulk offshore disposal. In contrast, the cross basin gradient for DDE points to PVS as a likely source and emplacement through cycle(s) of resuspension, advection and resettling. The extent and mechanisms of physical transport for DDT, DDD and DDE within SPB remain uncertain, but likely include adhesion to particulate matter and advection through mechanisms as modeled previously.^34^ Fourth, the widespread distribution of DDT throughout the deep SPB is remarkable insomuch as the pattern obviates the dumpsite paradigm locally, at least for bulk disposal of Montrose’s acid waste.

### 3.2 Environmental Variability

In addition to assessing the basin-scale distribution of DDT, DDD and DDE, we also conducted sampling targeted at the spatial variability of these compounds. In a comparison of two vertically resolved samples collected from the same nominal location (T1-a and A08, ∼260 m apart), notable differences are apparent in the maximum concentrations of DDT, DDD and DDE, although the stratigraphic patterns are similar (Figure S1). A second approach was also applied for all A-series stations, in which triplicate samples were collected in a vertically integrated manner (mini piston cores in Figure 4), revealing substantial spatial variability at a nominal distance scale of 5 cm (Figure 4). Observed variability was greater for DDT and DDD compared to DDE (Figure 4a-c), a distinction which is assumed to be driven by centimeter-scale depositional variability (e.g., caused by microtopography and particle-specific DDT loading) following pulsed disposal events during the ∼14-years of offshore disposal activity versus the protracted depositional history that followed for DDE. The observed centimeter-scale patchiness for DDT and DDD is surprising and reminiscent of the pattern for benthic oiling and oil biodegradation observed in the deep ocean following the Deepwater Horizon event.^35,36^

**Figure 4:**
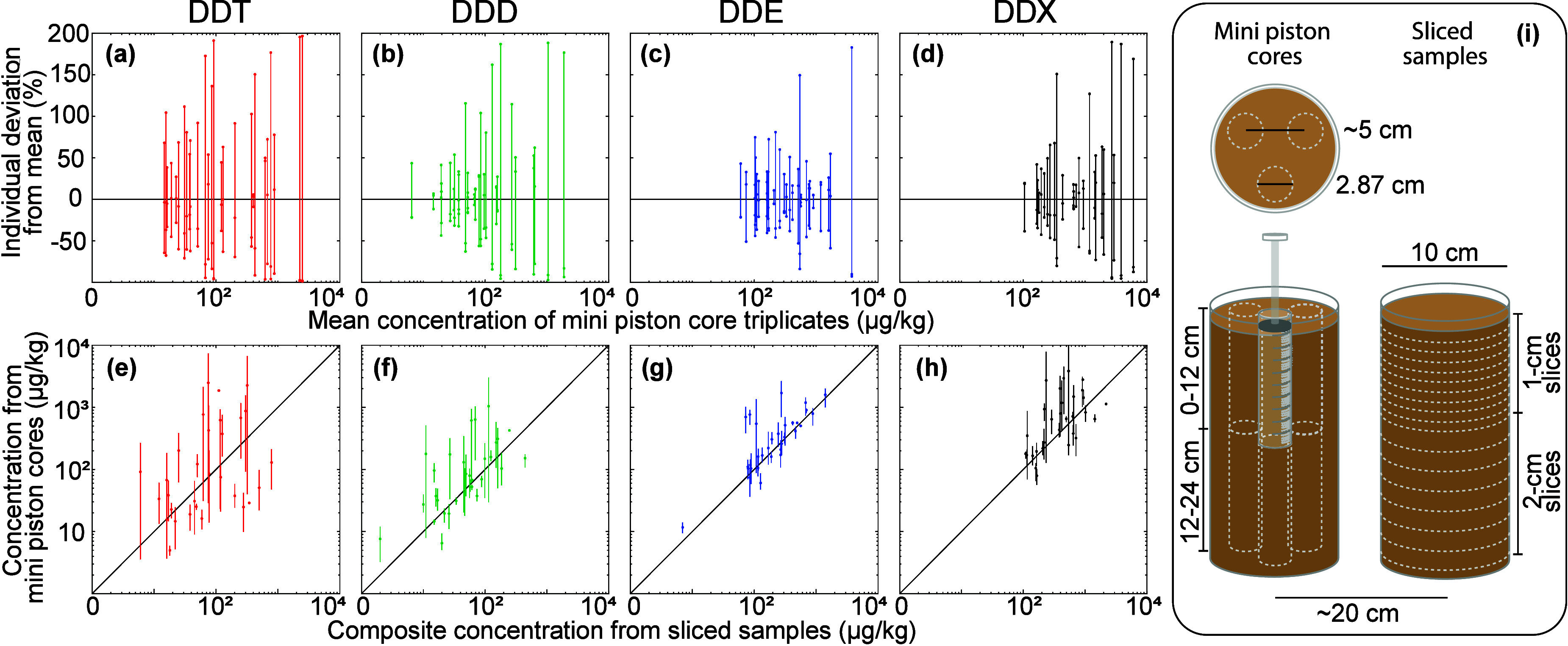
Variability of DDT, DDD, DDE, and DDX concentrations among mini piston core triplicates. Top row (a–d) shows percent deviation of individual concentrations of mini piston cores from triplicate mean concentrations (three dots connected by vertical bars), plotted against the triplicate mean concentration. Bottom row (e–h) shows concentrations of mini piston cores (dot: triplicate mean; vertical bar: range of concentrations) plotted against composite concentration reconstructed from sliced samples of corresponding depths. Note that *x*-axes of (a–h) and *y*-axes of (e–h) are based on a logarithmic scale. (i) Cartoon on the right illustrates the sampling scheme for mini piston core triplicates and sliced samples.

For each set of triplicate vertical mini piston cores collected, a parallel core was also analyzed through in-silico compositing of horizontal slices (sliced samples in Figure 4), comparison of which is displayed in Figure 4e-g. The horizontal composite approach integrates a surface area ∼12 times greater than each mini piston core sample and is thus taken to be more representative. Spatial variability is again clear in the comparison of these approaches, at a distance scale of 40–60 cm. The range of triplicate mini piston core values fails to encompass the value from the horizontal composite for roughly half the stations, for DDT and DDD, with substantially better agreement for DDE (Figure 4e-g). The underlying driver of this variability is unclear but could again be a combination of local scale accumulation patterns along with variability in the extent of dehalogenation experienced post deposition. Whatever the cause, local scale variability is substantial in this setting and is an important consideration for estimating sediment burdens, understanding infaunal exposure, and for interpretation of studies that rely on e.g., parallel collection of samples for DDT or DDD versus other parameters.

### 3.3 Sediment Inventories for DDT, DDD, and DDE

Local areal inventories (in mg m^–2^) for DDT, DDD, DDE, and DDX were calculated by integrating depth-resolved concentration measurements over the top 30 cm of sediment at each sampling location. Contour surfaces were generated from these areal inventories at each station using a natural neighbor interpolation surface with results displayed in Figure 5 for each analyte (and for each isomer in Figure S3). Spatial patterns for DDT, DDD and DDE inventories differ somewhat from the view of maximum concentration surfaces displayed in Figure 2, or temporal history displayed in Figure 3, with inventories approaching 100 mg m^–2^ for each compound. Notably, highest DDD inventory >100 mg m^–2^ appears northeast of dumpsite 2 while a high inventory (>200 mg m^–2^) of DDT resides in sediments located ∼10 km north of Catalina Island’s northwest tip. In contrast, DDE inventories approach 100 mg m^–2^ along the base of PVS and decrease gradually to ≤10 mg m^–2^ closer to Catalina Island. Strong spatial autocorrelation is observed for the DDT, DDD, DDE, and DDX inventories. Among them, DDE inventory shows the most gentle decline in autocorrelation values with increasing distance between sampling stations, consistent with the smoother data across space as shown in Figure 5c.

**Figure 5:**
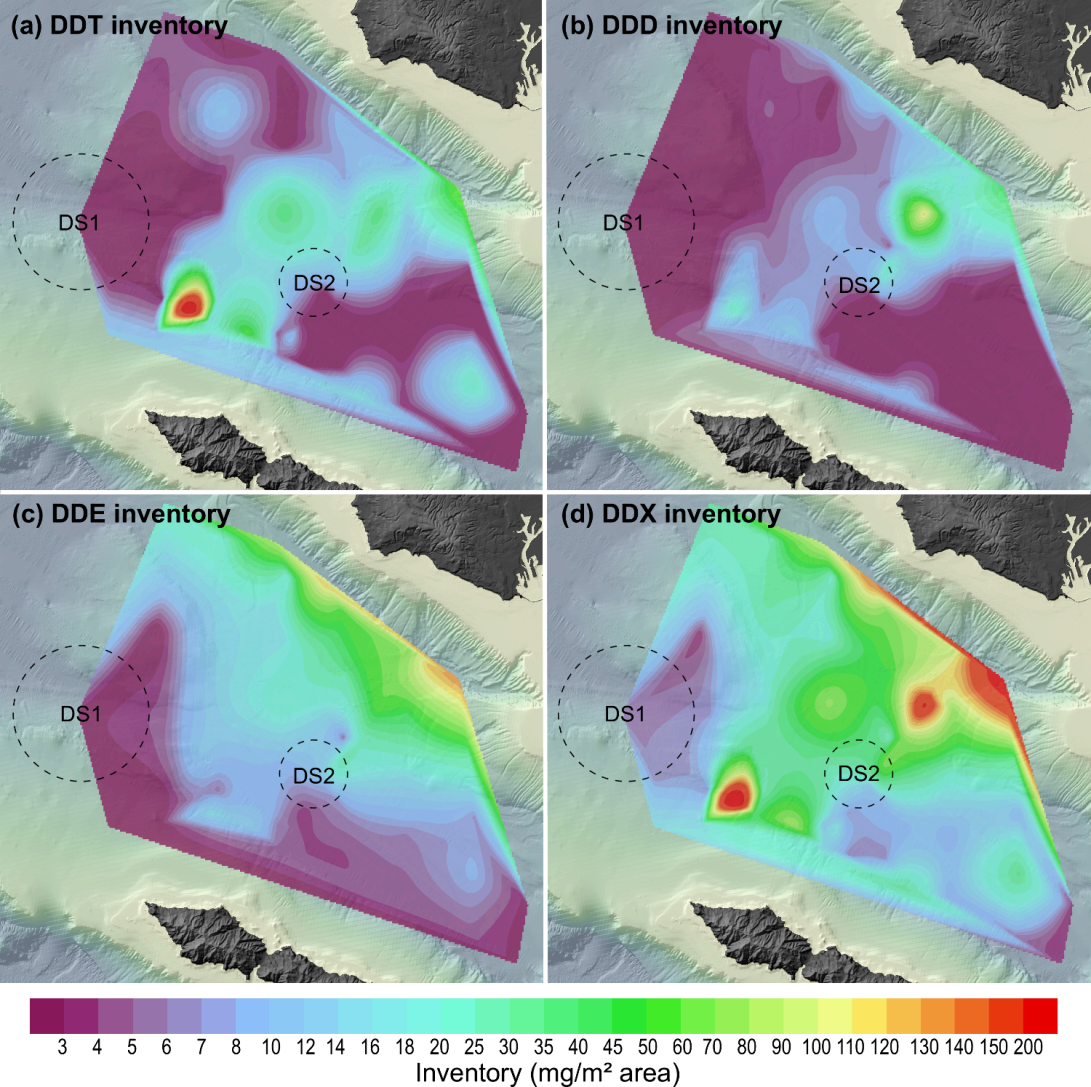
Spatial distribution of (a) DDT, (b) DDD, (c) DDE, and (d) DDX inventory integrated for the top 30 cm of sediments on a per square meter basis. Note that the color bar is on a nonlinear scale. Dashed circles indicate the locations of dumpsite 1 (DS1) and dumpsite 2 (DS2).

While the spatial distribution of DDX inventory enables a calculation of basin-scale burden, the observed environmental variability in concentration data at a range of spatial scales necessitates development of a statistical approach to estimate the associated uncertainty. Figure 6a and b illustrate how the uncertainty (standard deviation) in DDX inventory is evaluated following the jackknife-bootstrap approach described in Section 2.2. As expected, inventory estimation is better constrained as more stations are incorporated in each subgroup during the 1000 iterations of spatial integration. Using data from depth-resolved sediment slice samples from all stations and the jackknife-bootstrap approach for uncertainty, we estimated an average DDX inventory of 36.6 ± 4.4 mg m^–2^, scaling to a burden of 29.8 ± 3.6 tonnes DDX in the 814 km^2^ study area (9.4 tonnes DDT, 5.2 tonnes DDD and 15.2 tonnes DDE).

**Figure 6:**
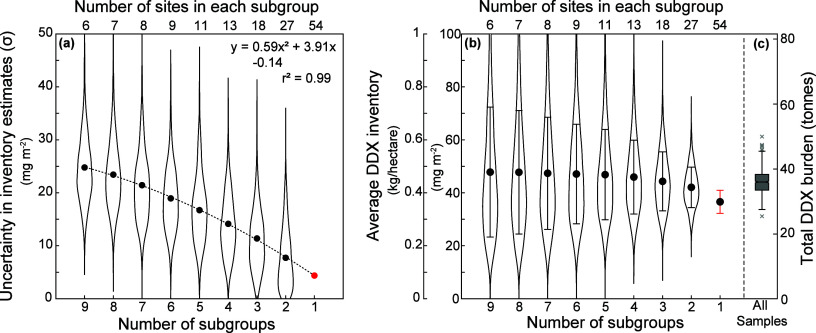
Evaluation of uncertainty and DDX inventory estimation. (a) Results from the jackknife-bootstrap approach illustrate the decline in uncertainty (standard deviation σ) of inventory estimates as the number of subgroups decreases. Violin plots show the distribution of σ from 1000 iterations with black dots showing the mean. The trend is fitted by a second order polynomial curve, which yields a mean uncertainty of 4.4 mg m^–2^ at *N* subgroup = 1 (red dot). (b) Violin plots showing the distribution of DDX inventory estimation from 1000 iterations with black dots showing the mean and error bars corresponding to σ shown in (a). (c) The uncertainty in average DDX inventory and total DDX burden estimation when centimeter-scale variability at each site is taken into account. Boxplot shows the distribution of results from 1000 iterations with the box representing the interquartile range (IQR), horizontal line in the middle representing the median, and whiskers representing data spread without outliers. Data beyond 1.5× IQR from the quartiles are considered outliers (gray crosses).

We further evaluated the uncertainty of our burden estimation by incorporating data from mini-piston core triplicates, which exhibit considerable centimeter-scale variability, in the areal inventory calculated for each site. Figure 6c displays the results from 1000 Monte Carlo iterations of spatial integrations, with each iteration randomly sampled from the uncertainty distribution at each site. The distribution of 1000 inventory estimations illustrate the uncertainty in our basin-scale DDX burden estimation when centimeter-scale uncertainty is considered for measurements at each site. By this approach we have estimated an average DDX burden of 44.2 ± 4.4 mg m^–2^, translating to ∼36 ± 3.5 tonnes DDX in the 814 km^2^ study area. These values are about 20% higher than the estimation based solely on depth-resolved samples. We attribute this discrepancy to the existence of centimeter-scale variability which frequently manifests as substantially higher DDX concentrations detected in one of the mini-piston core samples (as observed in Figure 4e-h) that skew the inventory estimates toward higher numbers in general. While acknowledging that consideration of centimeter-scale variability in our approach leads to added uncertainty in the basin-scale DDX burden estimation, we highlight that this represents a real challenge in attempting to estimate the burden of pollutants in an environment where concentrations are potentially highly heterogeneous at small spatial scales, such that the final result may be highly dependent on whether or not sampling has successfully captured hotspots.

The burden of buried DDX in SPB provides a useful lower limit for the discharge from offshore disposal, though actual disposal was likely much greater as our approach ignores deposition outside SPB, dechlorination beyond DDD or DDE, and e.g., mass loss associated with transformation of DDT to DDD and DDE. Prior estimates of offshore disposal are scant and rely heavily on the records of disposal volumes reported by Chartrand.^13^ Limited to a period in 1957–1958, Chartrand reported the disposal of 2000–3000 bbl per month (bbl = 159 L) of strong acid waste, which was then extrapolated to 230,000–330,000 bbl (37–53 million L) over an ∼14 year period from 1947 to 1961.^13^ Chartrand further estimated a 0.5–1% DDT content for the waste to yield a discharge estimate of 384–767 tonnes of DDT.^13^ The modern DDX burden in SPB from this work falls below the lower limit estimated by Chartrand, by a factor of more than 10, and the spatial pattern further suggests DDE contributions derived from PVS. While it is likely that substantial DDX deposited outside SPB and that the residues within SPB have been dehalogenated beyond DDD and DDE, it is also plausible that the assumptions in prior estimates led to overestimation of the total discharge.

### 3.4 Implications for DDT Waste Disposal Practices and Environmental Contamination

The extant spatial pattern of DDT in SPB provides useful insight as to the offshore disposal process as conducted by Cal Salvage starting in 1947 and allegedly terminating ca. 1961. Cal Salvage was alleged to have disposed of Montrose’s strong acid waste along with other wastes, e.g., from oil refineries, by pumping the waste directly from a barge into the surface water of the San Pedro Channel.^9,14^ The presence of an elevated DDT layer throughout SPB suggests that settling of the DDT occurred more quickly than complete dispersion by currents or dechlorination to e.g., DDE through biotic or abiotic means. However, distribution of DDT throughout SPB also points to advective transport during settling through the water column, with deposition lasting long enough to distribute DDT throughout the basin, but rapidly enough to manifest as cm-scale spatial patchiness. Furthermore, the elevated DDT located north of Catalina Island is consistent with a mechanism of deposition to shallower sediment of the Catalina slope followed by remobilization of DDT-bearing particles and transport by e.g., gravity flows through the submarine canyon system into the deep basin in the vicinity of station B06. The potential for deposition to sediments at shallower water depth near Catalina Island is a focus of ongoing investigation.

The spatial distributions of DDT and DDE in SPB carry three important implications. First, the modern concept of a DDT disposal site is flawed. To our knowledge there were no permits or designated sites when Cal Salvage began disposing of Montrose’s waste ca. 1947. Any dumpsite location was likely notional, operationally defined and decoupled with the reality of bulk waste disposal from a mobile platform or its subsequent environmental transport. Second, inferred transport of disposed DDT waste in the water column points to the likely possibility of transport outside of SPB and deposition to other settings. The extent to which such transport occurred is not presently known, in part because prior regional sampling efforts have focused on only surficial sediments shallower than 2 cm which are likely to miss the DDT-rich stratum associated with offshore disposal from the 1950′s.^37,38^ Third, a source of DDE into the deep SPB persists to the current time. We interpret this as transport of DDE-contaminated sediment from PVS, which is presumably accompanied by other contaminants including PCBs and other DDT-related compounds. The extent of water column contamination associated with shelf to basin transport and remobilization of near-seabed strata remains unknown.

## Supplementary Material







## Notes

Details on sediment density measurements; table of DDT family compounds quantified in this study; depth profiles of DDT, DDD, DDE, and DDX concentrations in each sediment core; contour maps of sediment accumulation thickness; spatial interpolation maps of DDT, DDD, DDE, and DDX isomer inventories; spatial autocorrelation of DDT, DDD, DDE, and DDX inventories (PDF)
Individual sample and inventory data (XLSX)
Heatmaps of decadal time steps (AVI)
