# Supplementary material for: Geospatial and Temporal Inventory for Industrial DDT Waste Disposal to a Deep Coastal Ocean Environment
Source: Environ Sci Technol. 2025 Sep 18;59(38):20578–87. doi: 10.1021/acs.est.5c03851 (PMC12490018; doi:10.1021/acs.est.5c03851)
Supplement: Supplementary file 1 [file es5c03851_si_001.pdf]

Supporting Information for:

Geospatial and Temporal Inventory for Industrial DDT Waste Disposal to a Deep Coastal Ocean Environment

Mong Sin Christine Wu[a], Jacob T. Schmidt[b], Hailie E. Kittner[b], Earth 182B Group[a], David L. Valentine[a,c*]

[a] Department of Earth Science, University of California, Santa Barbara CA 93106

[b] Interdepartmental Graduate Program in Marine Science, University of California, Santa Barbara CA 93106

[c] Marine Science Institute, University of California, Santa Barbara CA 93106

*Corresponding author: David L. Valentine

Email: valentine@ucsb.edu

Containing 7 pages, 1 table, 4 figures.

Text S1. Measurement of sediment density

Table S1. DDT family compounds quantified in this study

Figure S1

Figure S2

Figure S3

Figure S4

Reference

S1. Measurement of sediment density

Sediment % total solids was determined from subsamples of bulk sediment collected in the central San Pedro Basin aboard *R/V Yellowfin* in July 2023. Bulk sediment samples were stored at 4°C in glass jars with PTFE-lined caps following collection. Each 1 L jar was uncapped and stirred for ~5 minutes to homogenize thoroughly then subsampled in triplicate by syringe into glass scintillation vials with foil-lined caps. Triplicate samples were massed using an analytical balance then thoroughly dried in an oven at 61 °C until the change in consecutive daily mass measurements were <0.1 %, approximately 4 d. Dry mass was measured using an analytical balance and the % total solids were determined by mass ratio. All % total solids between replicates had <2% variability within the recommendations by EPA Method 1684.

A composite sample taken from 33° 37.866'N, 118° 21.960'W on July 27, 2023, was used to determine representative dry density for San Pedro Basin sediments. Replicate sediments dried using the methods above were ground using a mortar and pestle then re-dried under vacuum at room temperature to remove residual moisture from ~3 months of storage post initial drying. A subsample of each replicate was massed (n = 5 measurements per replicate) then analyzed for volume (n = 5 measurements per replicate) by He(g) displacement using a MicroMetrics AccuPyc 1330 pycnometer (TEMPO, University of California, Santa Barbara CA). Dry density was calculated for each replicate by a simple ratio of mass to volume, propagating measurement standard deviations. An average dry density considering all three replicates was reported as 2.5832 ± 0.0049 (1σ) g cm^{-3}. Since dramatic changes in sediment mineralogy across the deep SPB is considered unlikely, the measured dry sediment density, and an average seawater density of 1.025 g cm^{-3}, are used as basin-wide values in our subsequent calculation of site-specific wet sediment density based on average % solids of the top 30 cm sediment at each site. DDX areal inventories can then be calculated for the top 30 cm sediment based on DDX average concentration (on wet-weight basis), wet sediment density, and volume occupied.

Table S1. DDT family compounds quantified in this study.

IUPAC Name	Common Names
1-chloro-4-[2,2,2-trichloro-1-(4-chlorophenyl)ethyl]benzene	4,4'-DDT / p,p'-DDT
1-chloro-2-[2,2,2-trichloro-1-(4-chlorophenyl)ethyl]benzene	2,4'-DDT / o,p'-DDT
1-chloro-4-[2,2-dichloro-1-(4-chlorophenyl)ethenyl]benzene	4,4'-DDE / p,p'-DDE
1-chloro-2-[2,2-dichloro-1-(4-chlorophenyl)ethenyl]benzene	2,4'-DDE / o,p'-DDE
1-chloro-4-[2,2-dichloro-1-(4-chlorophenyl)ethyl]benzene	4,4'-DDD / p,p'-DDD
1-chloro-2-[2,2-dichloro-1-(4-chlorophenyl)ethyl]benzene	2,4'-DDD / o,p'-DDD
(Sum of all 6 compounds)	DDX

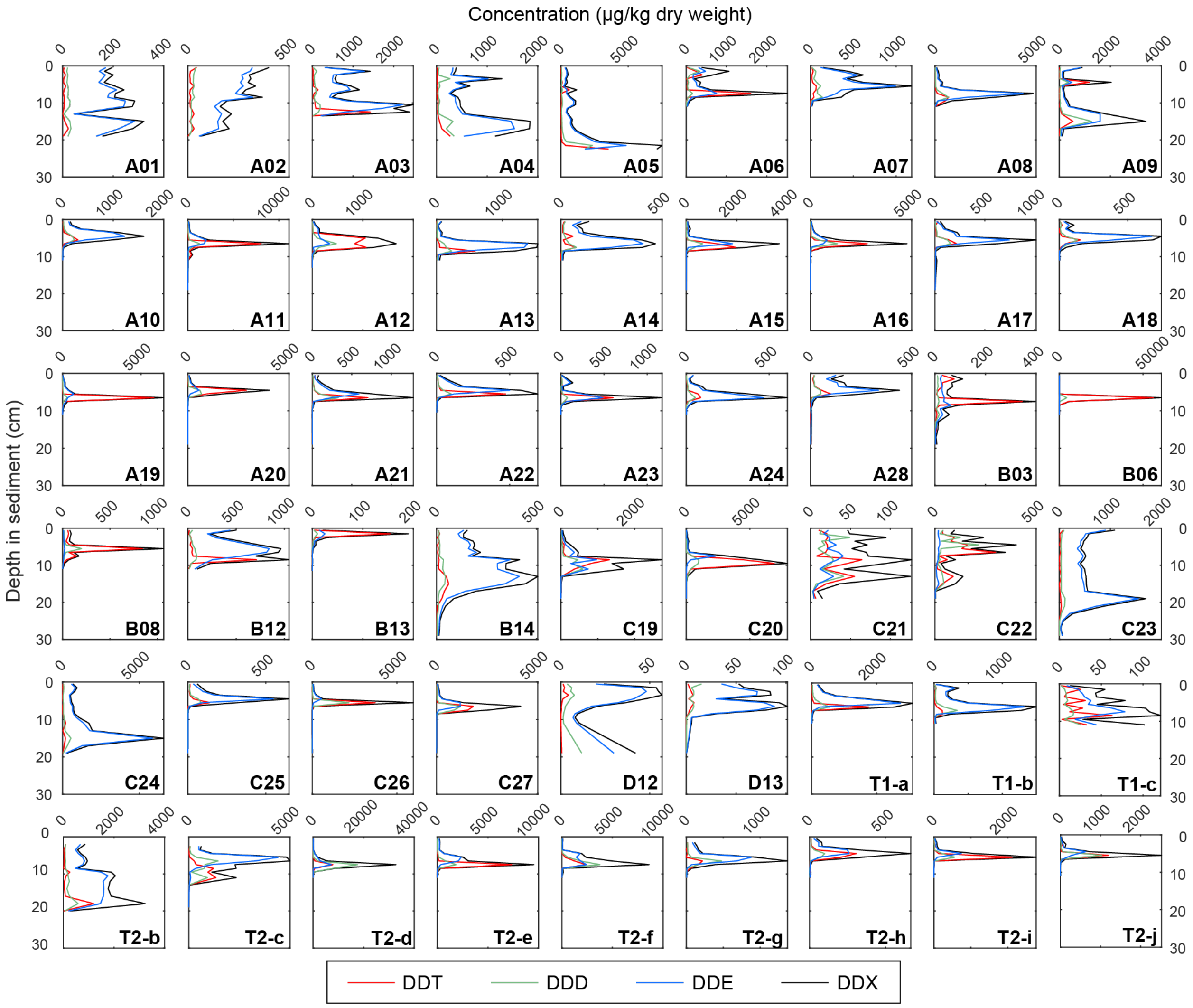

Figure S1. Depth profiles of DDT (red), DDD (green), DDE (blue), and DDX (black) concentrations in each sediment core. Profiles for all T stations (T1-a to c, T2-b to j) are from Schmidt et al., 2024.

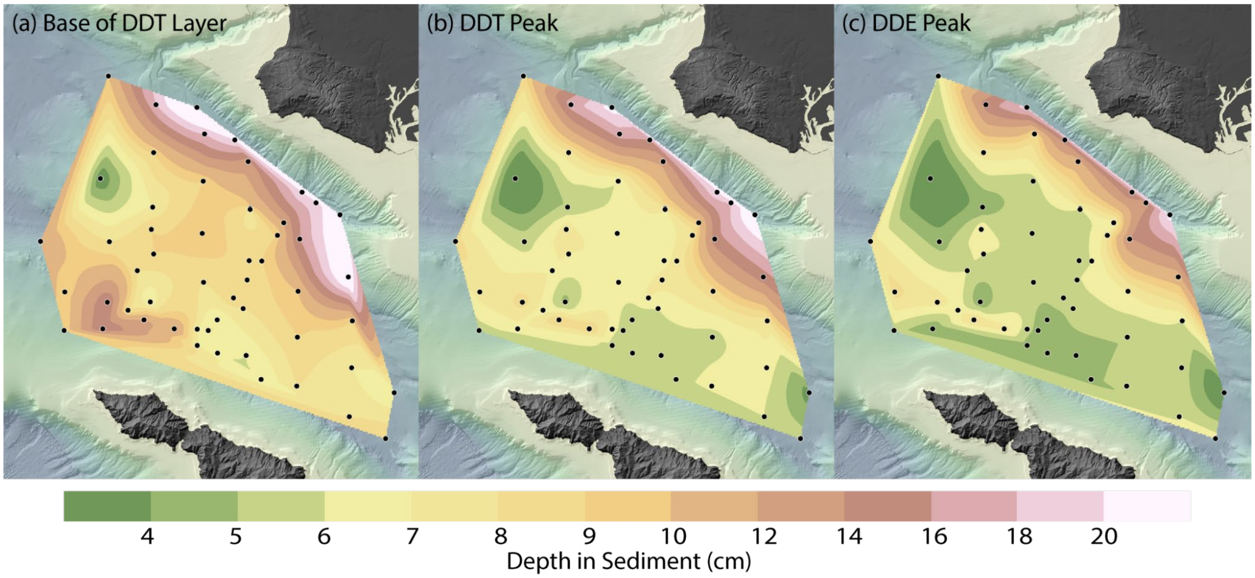

Figure S2. Contour maps showing the thickness of sediments accumulated since (a) the base of DDT layer, (b) DDT peak, and (c) DDE peak. The 'base of DDT layer' is determined as the depth below the main DDT horizon where DDT concentrations drop abruptly (to <2% of the peak DDT concentration). The depths of DDT peak and DDE peak are taken at the mid depths of the peak DDT or DDE horizon. The black dots indicate the locations of the sampling stations.

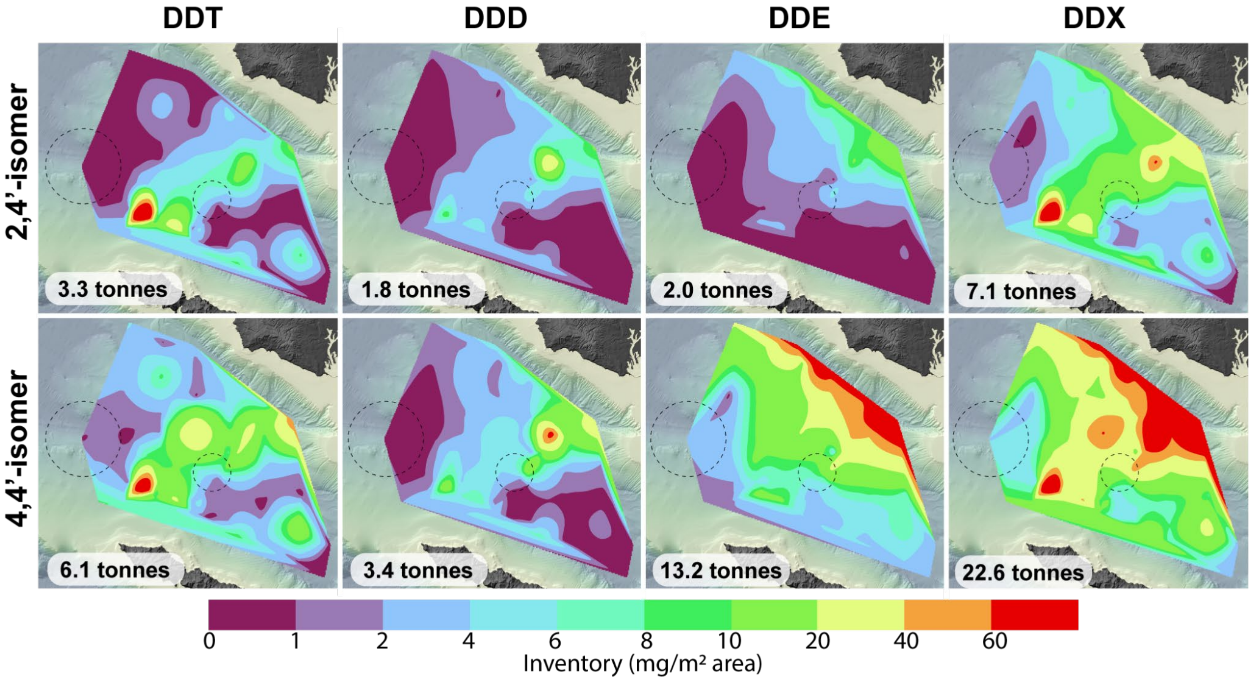

Figure S3. Spatial interpolation maps showing inventory of 2,4’- and 4,4’-isomers for DDT, DDD, DDE and DDX integrated for the top 30 cm of sediments. Numbers in tonnes show the estimated total burden for each isomer integrated over the 814 km^2 study area. The dashed circles indicate the locations of dumpsites 1 and 2.

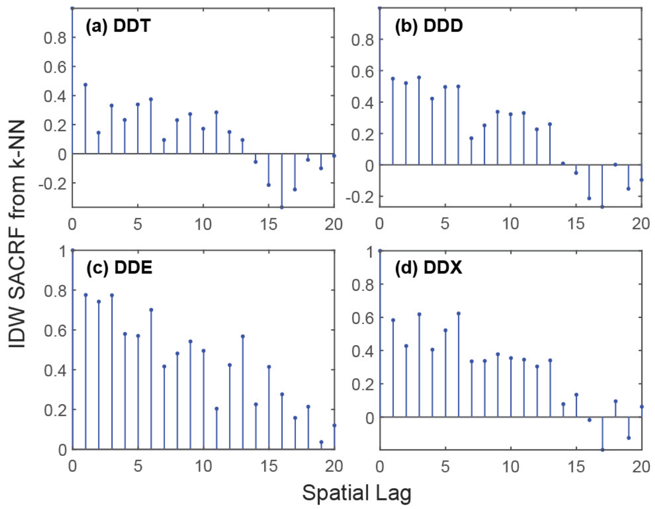

Figure S4. Illustration of the degree of spatial autocorrelation in (a) DDT, (b) DDD, (c) DDE, and (d) DDX top 30-cm inventories. Plots show the spatial autocorrelation functions (SACRF) with respect to the k-th nearest neighbors (k-NN) with inverse distance weighting (IDW), computed using a publicly available Matlab code (Grillenzoni, 2025). The high autocorrelation values at small spatial lag (close neighbors) suggests strong spatial autocorrelation, which gradually declines as distance increases. Among the three compounds, DDE displays the strongest spatial autocorrelation, and the gradual decline as distance increases suggests 'smoother' data across space.

Reference

Grillenzoni, C. Spatial Autocorrelation Function of a Process in the Plane (https://www.mathworks.com/matlabcentral/fileexchange/113695-spatial-autocorrelation-function-of-a-process-in-the-plane), MATLAB Central File Exchange. Retrieved July 2, 2025.
